# Understanding Mammalian Genetic Systems: The Challenge of Phenotyping in the Mouse

**DOI:** 10.1371/journal.pgen.0020118

**Published:** 2006-08-25

**Authors:** Steve D. M Brown, John M Hancock, Hilary Gates

**Affiliations:** University College London, United Kingdom

## Abstract

Understanding mammalian genetic systems is predicated on the determination of the relationship between genetic variation and phenotype. Several international programmes are under way to deliver mutations in every gene in the mouse genome. The challenge for mouse geneticists is to develop approaches that will provide comprehensive phenotype datasets for these mouse mutant libraries. Several factors are critical to success in this endeavour. It will be important to catalogue assay and environment and where possible to adopt standardised procedures for phenotyping tests along with common environmental conditions to ensure comparable datasets of phenotypes. Moreover, the scale of the task underlines the need to invest in technological development improving both the speed and cost of phenotyping platforms. In addition, it will be necessary to develop new informatics standards that capture the phenotype assay as well as other factors, genetic and environmental, that impinge upon phenotype outcome.

## Introduction

The phenotype of an organism is a complex product of its genetic constitution and various environmental influences. Determining and measuring phenotype has been a goal of all biologists, as the phenotype is an indicator of how an organism functions in different environments and under various challenges and insults. Comparative phenotyping underlies studies of evolutionary processes and how organisms adapt to their environment. Moreover, the phenotype is fundamental to understanding how genes function and how they determine both the developmental processes and the biochemical and physiological makeup of an organism. We cannot hope to understand an organism and the relationship between gene and phenotype without developing tools and techniques to study, measure, and annotate the phenotype.

Classically, geneticists have studied the relationship between genes and phenotype by the introduction of mutations in experimental organisms, the identification of novel phenotypes that arise from those mutations, and the investigation of the link between mutation and phenotype. In addition, population and evolutionary biologists study the relationships between changes in phenotypes between populations and species and underlying changes in allele frequencies. However, the issue of phenotyping has been thrown into focus by the establishment of programmes in several experimental organisms to systematically mutate every gene in the genome and determine the phenotype of each mutant. The aim of such programmes is to provide a systematic and comprehensive underpinning to a systems biology of these organisms where the relationship between mutant and phenotype is a key component to establishing a fundamental understanding of molecular and cellular processes. If we are to generate meaningful datasets that contribute to such an endeavour, it is clear that any determination of phenotype needs to be comprehensive. This sets apart these programmes from their predecessors where investigators focused on one or a few phenotypes of interest within their sphere of study. Geneticists are faced with developing approaches to phenotyping that encompass all developmental and physiological systems and that can be applied to a very large number of mutants. This will be a phenomenal undertaking in any organism, especially for the mouse.

## Mouse Models of Human Disease—Mutagenesis Programmes and Phenotyping

The mouse is the key model organism for the analysis of mammalian developmental, physiological, and disease processes [1]. It is the preeminent organism for the development and study of models of human diseases, and for many years it has not only provided insights into fundamental biological processes but also has played a major role in identifying genetic loci involved with disease susceptibility. A draft mouse genome sequence was published in 2002 [2], and a finished sequence will soon be available. The challenge for mammalian genetics in the 21st century is to build on the mouse sequence map and develop a comprehensive functional annotation of the mouse genome that will provide a rich resource for understanding human gene function. There is already available an extensive genetic toolkit to modify the mouse genome and to study the relationship between gene and phenotype. There are two distinct approaches to mouse mutagenesis—gene-driven and phenotype-driven [3,4]. In the former approach, a defined lesion is introduced into the mouse genome followed by investigation of its phenotype. Gene-driven approaches include gene-traps, targeted traps, and both targeted knock-out and knock-in mutations [5–8]. In contrast, the phenotype-driven approach aims to search large random collections of mutations, usually generated by ENU (ethyl-nitrosourea) mutagenesis, for phenotypes of interest irrespective of the underlying lesion generated [9–11]. Thus the phenotype is the starting point, and after discovery of an interesting phenotype the underlying genetic lesion is identified and investigated further. Both approaches depend upon phenotyping tools.

With the completion of the mouse genome sequence and the availability of powerful and affordable toolkits for mouse mutagenesis, there has been much public discussion of programmes to generate mutation resources for all mouse genes, principally using a range of gene-driven approaches [12,13]. Two such programmes, European Conditional Mouse Mutagenesis Programme and North American Conditional Mouse Mutagenesis Programme, focusing on generating libraries of embryonic stem cells carrying conditional mutations, are now funded and under way. It can be expected that additional programmes funded through the Knockout Mouse Project initiative in the United States will follow. ENU mutagenesis approaches, both phenotype-driven and gene-driven [14], will also make a significant contribution. Ultimately, comprehensive libraries of mouse mutants for every gene in the genome will be generated. Indeed, the goal of mouse geneticists is to have available a range of mutant alleles for every gene, including hypomorphs, gain-of-function, and dominant negatives, as well as null alleles. All of these mutations will require phenotyping. How do we achieve this and what are the challenges that face us in providing a comprehensive functional annotation of the mouse genome?

## The Challenges of Phenotyping

**Figure 1:**
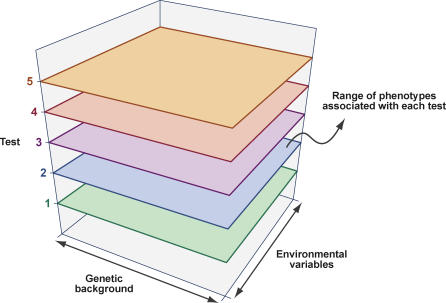
Phenotype Data Is a Complex Three-Dimensional Matrix of Information Comprising Phenotype Outputs from a Large Number of Tests on Individual Mutants in Defined Genetic Backgrounds under Specific Environmental Conditions

It is important to emphasise that phenotype is a complex output of gene allele, genetic background, environment, and the specific test applied. We can view phenotype as a complex matrix of these interacting factors (see Figure 1). For any gene, there will be multiple alleles to be investigated, potentially on multiple genetic backgrounds. The phenotype may be subject to many environmental influences, ranging from husbandry practices and cage environment to the pathogen spectrum. Finally, the phenotype test itself is a major contributing factor. How the test is performed may influence the measured output. In essence the ultimate goal of mouse genetics is to populate multiple matrices with phenotype data reflecting allele and genetic background, environmental conditions, and test details. The implication of this is that we need to standardise our approaches to how we undertake phenotyping. If mouse genetic centres around the world use various environmental conditions or adopt quite different test procedures, then much of the ensuing datasets will not be comparable. Importantly, we need to be able to share and compare datasets, allowing us to ask simple questions such as: Is the phenotype of allele 1 (measured in centre 1) the same as the phenotype of allele 2 (measured in centre 2); is the phenotype in allele 3 (measured in centre 3) different from the phenotype of allele 4 (measured in centre 4), or, trivially, was it simply the way the test procedure was implemented? We also need to be able to share relevant data with researchers concerned with the analysis of human disease, requiring us to work closely with clinical colleagues to design phenotyping experiments with this in mind. These simple questions indicate the depth of the problem and they mirror a wider concern in developing a systems biology of any organism, that is, the need for standardised datasets, appropriately annotated and underlined by uniform, systematic vocabularies [15].

### 

#### The fundamental role of the assay in standardisation of phenotyping.

To varying degrees, the phenotype assay is clearly crucial to determining the measured output. This would argue for adopting standardised approaches, or standard operating procedures (SOPs), for phenotyping. But to what extent do we need to ensure that assay and environmental conditions are monitored and as far as possible standardised to ensure comparability of datasets across time and place?

For some phenotypes, such as clinical biochemistry parameters, the output and its accuracy will depend on the equipment used (in this case an autoanalyser) and its inherent accuracy. Different machines may give different accuracies, underlining the need to record the equipment used. However, even in what are regarded as very standard measurements employing common equipment, other significant variables may contribute to the output, such as bleeding method, time of bleeding, diet, and so on. One study of the impact of caging and diet on various blood biochemistry parameters found little influence on phenotypes measured across a number of inbred strains [16]. However, it is just such evidence-based studies that are required to understand the inherent variables that must be controlled and to develop robust phenotyping platforms employing SOPs.

For other phenotype assays, there is substantive evidence that the SOP and environmental conditions are critical. Most notably, for complex behavioural tests, equipment design, equipment operation, and environmental conditions have a very significant impact on a test. For example, the design and operation of an open field can have a marked effect on test output. The position at which a mouse is introduced into an open field can have significant impact on test scores and thus on interlaboratory comparability. In a landmark study comparing behavioural test outcomes across a number of laboratories, Crabbe et al. found considerable variation between laboratories despite efforts to standardise procedures [17]. While the reasons remain unclear, it is possible that unrecognised factors, in test or environmental procedures, contributed to the variation. It would be wrong to conclude that behavioural testing is fraught with difficulties, rather that even greater efforts are needed to examine the causes of variation in test procedures. Indeed, the Eumorphia Project (European Union Mouse Research for Public Health and Industrial Applications) (http://www.eumorphia.org), a collaborative research programme funded by the European Commission, has made a major effort to standardise and validate procedures for behavioural tests both within and between laboratories [18]. While many tests were validated, others showed considerable variation in test output between laboratories and require further examination and elimination of test variables.

As might be expected, behavioural tests may be subject to multiple environmental influences, including cage housing and environmental enrichment. One recent study by Wolfer and colleagues [19] investigated the effect of enrichment on the variance contributed by strain x laboratory interactions. They demonstrated that enrichment effects were highly consistent across laboratories and dismissed concerns that enrichment might disrupt standardisation. However, intriguingly, they also found that environmental enrichment had little influence on strain ranking in their response to several behavioural tests. This study, though, underlines the difficulties we face. Only two strains were used in the Wolfer study and inevitably not all available behavioural tests were examined. By contrast, the Eumorphia Project, employing a wider set of inbred strains and additional tests, found that environmental enrichment clearly affected strain ranking for some inbred strains under several behavioural tests [20]. For other tests, there were no changes in strain ranking.

In conclusion, it is important to catalogue both the assay and the environmental conditions in which the test was performed. Moreover, we can conclude that adopting SOPs and as far as possible adopting common environmental conditions will assist in the population of comparable datasets of phenotypes. We discuss below what progress is being made towards delivering common, standardized phenotyping platforms.

#### The importance of throughput—Hierarchies of tests.

**Figure 2:**
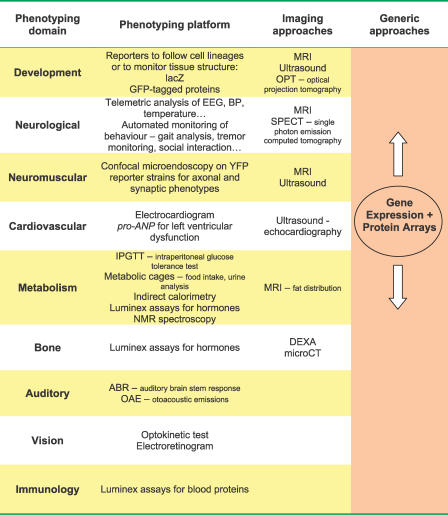
Examples of New Phenotyping Platforms and Imaging Approaches Which Are Beginning to Be Applied, or Being Considered, for High Throughput Mouse Phenotype Screens on a Large Number of Mutant Animals The table is not comprehensive but provides exemplars of tests where technological developments are providing improvements in speed and cost that will enable their utilisation in broad primary screens of mouse mutants. Note that there is increasingly the potential for generic molecular phenotyping approaches such as gene expression and protein arrays that impact upon multiple phenotyping domains to be applied as primary screens.

Given the numbers of mutant mice that will need to be tested to develop a complete functional annotation of the mouse genome, there has been considerable emphasis on developing tests with high throughput that can be used to rapidly screen large numbers of mice. To date the prevailing wisdom has been to employ a hierarchical approach to phenotyping whereby in the first instance rapid, comprehensive test batteries, often utilising relatively unsophisticated tests, are applied to large numbers of mice—so-called *primary* screens. Primary screens provide a superficial but broad assessment of mouse phenotype. Subsequently, mice identified with potential phenotypes of interest are the focus of more time-consuming, in-depth sophisticated tests—the *secondary* and *tertiary* screens (see Figure 2). A number of test batteries have been proposed that employ this hierarchical approach focusing either on specific functional domains, e.g., behaviour [21,22], or employing a wider selection of screens, e.g., SHIRPA (SmithKline Beecham, Harwell, Imperial College, Royal London Hospital Phenotype Assessment) [23], a comprehensive phenotype assessment tool involving a battery of up to 40 simple tests (http://www.mgu.har.mrc.ac.uk/facilities/mutagenesis/mutabase/shirpa_summary.html). Clearly, there are not fixed boundaries between primary and secondary/tertiary screens and, depending upon investment and resources, even quite sophisticated tests can be employed as primary screens.

#### Bringing technology to phenotyping—Speed and sophistication.

The challenge for the future is to reverse the traditional inverse relationship between throughput and sophistication. This is particularly important if we recognise that the phenotype description of a mutant mouse needs to be both comprehensive and deep if we are to fully understand gene function and integrate it with various hierarchical systems descriptions at cellular and physiological levels. Clearly we can address this challenge by investment in new technology bearing on equipment and test design that brings speed and sophistication to the phenotype platform, as well as improvements in cost (see Figure 2). Even quite complex behavioural assays, such as circadian rhythm and sociability, have benefited from elaborate automation and data capture and can be utilised as primary screens [24,25]. In the future, we can expect microtechnologies and remote monitoring to have a significant impact on the speed and flexibility of phenotype screens. Already, new technologies such as Luminex are set to revolutionise the rapidity and scale of assaying a wide spectrum of blood proteins [26].

In contrast, some areas of phenotype analysis, such as pathology or gene expression microarray analysis, for the moment remain inexorably fixed as a secondary or tertiary screen, given the difficulties of automating tissue and section analysis from a large number of organs and the requirement for expert assessment of each and every stained section [27,28]. The importance of pathology in mouse phenotyping cannot be underestimated. However, the laborious nature of pathology analysis and the dependence on a small cadre of experts continues to represent a significant stumbling block to unravelling the mouse phenome.

#### Controlling for phenotype variation—Controls and genetic background.

In assessing phenotypes, the issue of controls and genetic background deserves some attention. It is common practice to cross mutants generated in 129 embryonic stem cells onto a C57BL/6 background. Indeed, the C57BL/6 background is the preferred background for phenotype analysis in a number of systems, including neurology, behaviour, and immunology. However, relatively few mutants are backcrossed sufficiently (ten or more generations) to develop congenic lines for which C57BL/6 mice would act as appropriate controls. Generally, control mice are wild-type sibs generated from the same intercrosses used to generate homozygous mutant mice, and both mutant and wild-type mice will contain segregating portions of 129 and C57BL/6 genomes. It is possible that this has led to some of the variation seen in mutant phenotypes both within and between laboratories. However, if, as seems likely, mutants will be produced in a new generation of C57BL/6 embryonic stem cell lines, it will be possible to readily create all mutations on a pure inbred C57BL/6 background, avoiding the current pitfalls. If this can be done, what of the necessary controls? Undertaking the necessary heterozygous intercrosses on the shelf to generate sufficient age-matched homozygotes and wild-type sibs for phenotype analysis is both time-consuming and costly. With the advent of in vitro fertilisation techniques in the mouse [29], it would be straightforward and efficient in the case of viable mutants to generate large numbers of age-matched homozygotes for analysis by in vitro fertilisation. At the same time, a rolling baseline of wild-type phenotype data could be produced by intermittent analysis of the C57BL/6 background strain. Economies such as these will be necessary, given the phenomenal workload that will be required to analyse the tens of thousands of mutants over the coming years.

## Landmarks in Phenotyping

In addressing the challenges of mouse phenotyping, it is worth cataloguing some landmarks along the road, several of which point us towards future directions.

### 

#### Irwin, the SHIRPA screen, and other primary screens.

In 1968, Irwin [30] developed one of the first primary screens for assessing pharmacological and toxicological responses. This was adapted to develop a general primary screen for mouse mutant phenotypes called SHIRPA [9,23]. It consists of a battery of about 40 simple tests, subsets of which address deficits in spinocerebellar, autonomic, neuropsychiatric, neuromuscular, and sensory function. SHIRPA was successfully used as a primary screen in one of the first major phenotype-driven ENU mutagenesis programmes to identify potential mutant phenotypes of interest [9]. It has been used subsequently, usually in a modified form, in other ENU mutagenesis programmes, as well as being employed as a general screen for the primary phenotypic characterisation of mutant mice [31]. At the same time as SHIRPA was developed, attention was being given to the development of hierarchical screens that delivered phenotyping platforms for specific systems, including behaviour [21]. These first attempts to develop systematic, robust phenotyping screens are the forerunner of later efforts to develop more comprehensive, validated sets of primary screens, such as EMPReSS (European Mouse Phenotyping Resource for Standardised Screens) (http://www.empress.har.mrc.ac.uk/), a database of phenotype screens that can be used to phenotype a mouse.

#### The Mouse Phenome Project.

In 2000, the Jackson Lab began a programme—the Mouse Phenome Project—initiated by Ken Paigen to develop a comprehensive database of phenotypes from the mouse inbred strains [32,33] (http://www.jax.org/phenome). The project did not envisage an organised programme of mouse phenotyping either locally or distributed. The main intention was to provide a database for the collation and dissemination of phenotypes developed on sets of mouse inbred strains collected by diverse research groups. The Mouse Phenome Project encouraged research groups to contribute and make available their data through the Jackson Lab portal. The database provides several tools for downloading and viewing the phenome data. Crucially, the Mouse Phenome Project recognised the importance of baseline phenotypes of inbred strains as a key underpinning for mouse genetics studies and the key value of systematic phenotype information.

#### The Eumorphia Project.

In 2003, a consortium of European laboratories began a programme to develop standardised phenotyping platforms that allow reproducibility of test outcome over time and place [34], and in so doing address one of the key challenges for the future of phenotyping. The consortium comprises 18 research institutes working on establishing and validating new phenotyping methods. Eumorphia has developed a new robust primary screening protocol, EMPReSS. EMPReSS incorporates more than 150 SOPs and associated annexes and appendices, many validated on a cohort of inbred strains across a number of laboratories. EMPReSS SOPs are available for all the major body systems, and also include SOPs for generic approaches such as imaging, pathology, and gene expression. Ultimately, the community needs to build on the Eumorphia programme, enlarging the resource of available SOPs to secondary and tertiary tests and to provide a comprehensive resource of standardised and validated screens, bringing further comparability and reproducibility to phenotyping platforms. It will be important as mouse genetics centres around the world begin the task of phenotyping the many mutant lines emerging from the European Conditional Mouse Mutagenesis Programme, North American Conditional Mouse Mutagenesis Programme, and Knockout Mouse (a collaborative research project to be funded by the NIH) projects, as well as recombinant inbred strains such as those developed by the Complex Trait Consortium [35], that standardised phenotyping platforms such as EMPReSS or others are employed.

#### The Mouse Clinic—The future of mouse phenotyping.

Even with rapid, standardised protocols applicable to diverse cellular and physiological systems, there is still a phenomenal mountain to climb to complete the phenotyping of mutant alleles for every gene in the genome. Moreover, it is unrealistic to expect many laboratories to have available the expertise or the equipment and infrastructure to carry out comprehensive phenotyping of mutant alleles of local interest. Consequently, the concept of mouse clinics has emerged. There are a number of mouse genetics institutes around the world that have broad expertise in phenotyping and can be classified as phenotyping centres. A few of these have adopted the clinic concept and offer their phenotyping expertise to external users—these include the German Mouse Clinic (http://www.empress.har.mrc.ac.uk/) at the GSF National Research Centre for Environment and Health in Munich (http://www.gsf.de/ieg/gmc/) [36], and the Mouse Clinical Institute—Institute Clinique de la Souris in Strasbourg (http://www-mci.u-strasbg.fr/index.html). It is clear that if we are to accomplish a comprehensive functional annotation of the mouse genome, we will need to develop additional phenotyping centres if we are to generate the scale of infrastructure that will meet the inherent phenotyping demands. Many of these phenotyping centres would undoubtedly operate as clinics, offering services to the wider community as well as being centres for phenotyping development. It is important to emphasise that it is likely that these clinics will be at the centre of distributed networks of phenotyping able to call on smaller centres with the specialist expertise to carry out more sophisticated experiments in a particular area. By working together, the clinics and these more specialised centres will collectively push the boundaries of phenotyping technology and application.

## Information Systems for Phenotyping—Ontologies and Databases

As discussed above, we cannot expect to observe a simple one-to-one relationship between individual mutations and individual phenotypes. Rather, we must expect the mapping from genotype to phenotype to take place via complex patterns of genetic interactions and environmental conditions (systems biology). Except in the simplest of cases, systems analysis will require the use of computational tools to infer patterns of interaction and model genotype–phenotype relationships. To make best use of computational systems biology approaches, it will be necessary to develop sophisticated ways of representing phenotype data and easily accessible databases of phenotype information related to genetic background and other relevant information.

Representing phenotypic information in a standardised manner presents new challenges for bioinformatics, which has traditionally concerned itself with simpler data types (DNA and protein sequence for example; it is worth remembering that even these simple data types pose immensely difficult problems of interpretation, for example in gene and protein structure prediction). A significant breakthrough in the representation of complex biological entities in computational biology came with the introduction of ontologies into bioinformatics [37] and in particular with the advent of the Gene Ontology (GO) [38]. The Gene Ontology Consortium contains representatives of a number of model organism databases, including the Mouse Genome Database at the Jackson Laboratory. It was a logical step forward to consider the development of similar ontological structures for the representation of phenotypes. A straightforward approach to mouse phenotype information is taken in the Mammalian Phenotype Ontology [39] (http://www.informatics.jax.org/userdocs/mp_browser_help.shtml), which is curated at the Jackson Laboratory. However, as we have noted already, the exact details of the phenotyping protocol, genetic background, and environmental conditions are likely to play a significant role in determining the observed phenotype, and this is not yet taken into account in the Mammalian Phenotype Ontology. A more recent development is to use a compound description of phenotype built up from component ontologies that allows these other features to be represented [40]. In this approach, the assay used to ascertain the phenotype information is central, thus explicitly recognising its importance in descriptions of phenotype. This combinatorial approach has another significant advantage in that it opens the door to assigning mice or mouse strains with ontological descriptors, using the assay as a point of entry to the annotation process: SOPs need only be annotated once with an ontological description of what they measure to allow data to be annotated with the same description. There are still significant issues to be addressed before ontologies of this kind can be wholly satisfactory, however. For example, frameworks for the description of environmental conditions as well as a more complete structure for the description of pathological states remain to be developed. Further, there are significant theoretical issues, such as how to deal with data that only indirectly measure phenotypic characters of interest, such as the results from many behavioural experiments.

A second important issue is the availability of raw phenotyping data. Just as expression levels of different genes show patterns of correlation, it seems likely that individual phenotypic characters will show correlations resulting from common underlying processes. Mining for such relationships, especially in the context of genetic differences, would require the availability of large databases of raw, rather than digested, data. At present, two databases present raw baseline data for specified genetic backgrounds (inbred mouse strains): the Mouse Phenome Database [32] (http://www.jax.org/phenome) and EuroPhenome Database (http://www.europhenome.org), a database that contains the phenotype information derived from EMPReSS SOPs. Standardised SOPs for phenotyping are available from the Mouse Phenome Database and the EMPReSS collection developed by Eumorphia [41] (http://www.empress.har.mrc.ac.uk/). The Mouse Genome Database [42] contains a considerable amount of information on mutant strains annotated using the Mammalian Phenotype Ontology (http://www.informatics.jax.org/searches/MP_form.shtml), but this is summary information, extracted largely from the literature, and does not contain information on individual mice which would be useful for assessing variability in a given strain. Some of the larger mouse phenotyping centres make strain data available via their Web sites, but as there is no consistent format for data representation or exchange, these data are also not readily accessible for data mining. More progress will need to be made towards either an integrated database of phenotype information or, more likely, standards for data representation and exchange that will facilitate downstream analysis.

## Conclusion

The challenges in phenotyping awaiting mouse geneticists as they contemplate the systematic mutagenesis of the mouse genome are enormous. Overcoming these challenges will first require significant technological developments that will improve both the speed (and cost) as well as the sophistication of phenotyping measures. Second, providing phenotype datasets that can be shared and compared will require continuing efforts to implement standardised phenotyping protocols and to understand the variables both in the testing process and the environment that impinge upon output. Last, we need to continue to develop new standards for phenotype data representation that incorporate the phenotype test as well as other variables. The next step is to begin to apply these requirements in pilot projects that undertake the systematic phenotype analysis of a significant number of mutants from the new global genome-wide mutagenesis programmes.

## Abbreviations

EMPReSS: European Mouse Phenotyping Resource for Standardised Screens
ENU: ethyl-nitrosourea
Eumorphia: European Union Mouse Research for Public Health and Industrial Applications
SHIRPA: SmithKline Beecham, Harwell, Imperial College, Royal London Hospital Phenotype Assessment
SOP: standard operating procedure
